# Mental Health among Spanish Adults with Diabetes: Findings from a Population-Based Case–Controlled Study

**DOI:** 10.3390/ijerph18116088

**Published:** 2021-06-04

**Authors:** Marta Lopez-Herranz, Rodrigo Jiménez-García, Zichen Ji, Javier de Miguel-Diez, David Carabantes-Alarcon, Clara Maestre-Miquel, José J. Zamorano-León, Ana López-de-Andrés

**Affiliations:** 1 Nursing Department, Faculty of Nursing, Physiotherapy and Podology, Complutense University of Madrid, 28040 Madrid, Spain; martal11@ucm.es; 2 Department of Public Health & Maternal and Child Health, Faculty of Medicine, Complutense University of Madrid, 28040 Madrid, Spain; dcaraban@ucm.es (D.C.-A.); josejzam@ucm.es (J.J.Z.-L.); anailo04@ucm.es (A.L.-d.-A.); 3 Respiratory Care Department, Health Research Institute of the Hospital General Universitario Gregorio Marañón (IiSGM), Complutense University of Madrid, 28007 Madrid, Spain; jizich72@gmail.com (Z.J.); javier.miguel@salud.madrid.org (J.d.M.-D.); 4 School of Health Sciences, University of Castilla–La Mancha, 45600 Talavera de la Reina, Spain; Clara.Maestre@uclm.es

**Keywords:** diabetes mellitus, mental disorders, psychological distress, psychotropic drugs, prevalence

## Abstract

Background: The purpose of this study was to assess and compare the prevalence of self-reported mental disorders, psychological distress, and psychotropic drug consumption among people with diabetes mellitus (DM) and matched non-DM controls. Methods: A case–controlled study using data from the Spanish National Health Interview Survey was conducted in 2017. We identified 2116 DM adults (aged ≥35 years). Non-DM controls were matched 1:1 by age, sex, and province of residence. Results: Prevalence of mental disorders (25.0% vs. 19.2%; *p* < 0.001), psychological distress (29% vs. 19.5%; *p* < 0.001), and consumption of psychiatric medications (29.7% vs. 23.5%; *p* < 0.001) among DM cases were higher than those among matched non-DM controls. The DM patient variables associated with experiencing a mental disorder, psychological distress, and consumption of psychiatric medications were: being a woman, worse self-rated health, and a visit to a psychologist within the last year. Older age (≥80 years) was associated with a lower probability of reporting mental disorders and psychological distress among DM cases. Not practicing physical exercise was significantly associated with experiencing psychological distress. Conclusions: Adults with DM included in our investigation have a significantly higher prevalence of mental disorders, psychological distress, and consumption of psychiatric medications than non-DM controls. It is necessary to implement screening strategies and psychological interventions to improve the mental health of DM patients in Spain, focusing especially on women and those aged 35 to 59 years.

## 1. Introduction

Diabetes mellitus (DM) is a chronic illness associated with an increased risk of mental disorders, the most common being anxiety and depression [1]. High-quality studies, including systematic reviews, have reported a higher prevalence and incidence of mental disorders in people with DM compared with those without this condition [2,3,4,5,6]. Lin et al. [5] found that people with DM have a higher risk of depression (OR 1.38 95% CI 1.15–1.66) and anxiety (OR 1.20; 95% CI 1.01–1.42) than those without DM. Furthermore, among people with DM, mental disorders increase physical and mental impairment and are associated with a lower adherence to DM treatment, worse glycemic control, more diabetic complications, a diminished quality of life, and increased disability [4,6,7,8,9].

Psychological distress is frequently found in people who experience DM and has been associated with adverse health outcomes and unhealthier behaviors [10,11]. Gomez-Pimienta et al. [12] found that around 32% of patients with DM presented high diabetes-related emotional distress and had a decreased quality of life. 

Previous studies have suggested a bidirectional association of pharmacologic treatment for various psychiatric disorders and DM [13]. In this regard, the consumption of psychotropic drugs in patients with type 2 diabetes has been reported to lead to a higher use of emergency services and hospitalizations [14].

Previous investigations have confirmed that, in individuals with type 1 or type 2 diabetes, depression, anxiety, or even DM distress below the threshold for a psychiatric diagnosis negatively impact diabetes treatment and control and thus result in more poorly managed diabetes [15]. 

DM distress has been described as emotional reactions that appear as a result of living with DM. DM distress is frequently misdiagnosed and has been associated with suboptimal self-care, poorer glycemic control, and an increased risk of anxiety and mood disorders [16,17,18].

The increase in the incidence of DM in recent years and the association of this condition with mental disorders is expected to result in a significant increase in the social and medical costs of DM in the future [19]. 

In Spain, a previous study using the 2006 Spanish National Health Interview Survey (SNHIS) found that people with diabetes reported mental disorders and psychological distress more frequently than those without diabetes. These differences remained significant after multivariable adjustment [20].

The aims of the current investigation were, using the SNHIS conducted in 2017 (SNHIS2017), to: i) assess the prevalence of self-reported mental disorders, psychological distress (GHQ-12 ≥ 3), and consumption of psychiatric medications among persons experiencing DM; ii) compare these prevalence with sex-and-age-matched non-DM controls; and iii) identify which sociodemographic and health-related variables are associated with reporting mental disorders, psychological distress, and consumption of psychiatric medications among persons with DM.

## 2. Materials and Methods

### 2.1. Study Design and Study Population

We conducted an epidemiological retrospective observational case–control study. The data for our investigation were obtained from the SNHIS2017. Details on the methodology of the SNHIS2017 are described elsewhere [21,22]. 

The SNHIS2017 was designed to provide reliable estimates of the population living in Spain and aged 15 years or over and included a total sample size of 23,089 participants. The information collection period was October 2016 to October 2017. Computer-assisted personal interview was the method used to collect the information. Given the low prevalence of DM in Spain among those aged under 35 years, we included only subjects aged ≥35 years in our investigation [23]. The total number of respondents with DM aged under 35 years was 20 (<1%).

### 2.2. Case–Control Design

Subjects were classified as DM cases if they reported experiencing DM and the diagnosis of this condition had been confirmed by a physician. Subjects who answered “no” to the presence of DM were included to create the control group. 

For each case, we selected a non-DM control matched by sex, age, and province of residence. If more than one control was available per case, the selection of the case was done randomly. We found a control for almost all DM individuals (98.6%).

Details in the questions used to create our study variables can be found in Table S1 and in the SNHIS2017 methodology [21,22].

### 2.3. Study Variables

We measured the mental health using three dependent variables:

(1) The self-reported presence of a “mental disorder” was defined as the person interviewed reporting depression and/or anxiety, with these conditions being diagnosed by a medical doctor.
(2) The presence of “psychological distress” was assessed using the 12-item General Health Questionnaire 12 (GHQ-12). The Spanish version of the GHQ-12 has been validated and a cutoff of 3 was recommended to identify individuals with psychological distress in previous investigations [20,24,25].
(3) The variable “consumption of psychiatric medications” was created using questions regarding the self-reported use of physician-prescribed medications in the last two weeks. We considered psychiatric medications as any of the following: “tranquilizers (anxiolytics),” “sedatives (anxiolytics),” “sleeping pills (anxiolytics),” and/or “antidepressants”. In Spain, the terms “*tranquilizers*” and “*sedatives*” are used indistinctly by people from outside the healthcare industry to refer to anxiolytics.

Independent variables included: (i) sociodemographic characteristics such as sex, age, living with a partner, education level, and social class; (ii) health-related variables such as self-rated health,; use of healthcare services in the last year (emergency services, hospital admission, visit to psychologist); (iii) self-reported presence of doctor-diagnosed concomitant chronic diseases (hypertension, heart disease, arthrosis, stroke, DM, malignant tumors, chronic pain, and/or permanent injuries); and (iv) lifestyle-related variables (obesity, alcohol consumption, current smoking habit, and physical inactivity). Descriptions and categories for these variables are shown in Table S1.

We analyzed the variable “living with a partner” (Table S1) instead of “marital status” because the objective of selecting this variable was to assess whether living alone, given the social and emotional support provided by a partner, is associated with mental health variables, as suggested by other authors [26,27,28].

Shown in Tables S2–S4 are the distribution of the mental health variables among those with and without DM to show those who reported one, two, or three of the different parameters that are used. As can be seen in these tables, most subjects with DM only had one of the three parameters analyzed (44.8%), with 31.6% presenting two and 23.5% presenting three (Table S2). The equivalent figures for the non-DM controls were 48.1%, 32.9%, and 19.0%, respectively (Table S2). The most frequent association was the consumption of psychiatric medications among those reporting mental disorders, found in 72.5% of people with DM and 71.2% among the controls (Tables S3 and S4).

### 2.4. Statistical Analysis

The distribution according to the independent study variables was described and compared for DM cases and matched non-DM controls. The statistics used for description included absolute frequencies and proportions for prevalence. To compare prevalence between cases and controls, bivariate conditional logistic regression models were applied. If the participant answered “don’t know” or “prefer not to answer,” the case and control were excluded for the analysis of that variable.

To assess the association of study variables with the presence of mental disorders, psychological distress, and consumption of psychiatric medications among DM cases, we constructed three unconditional logistic regressions following Hosmer et al.’s recommendation [29]. We included in the multivariable models all the independent variables with significant bivariate associations (*p* < 0.10), with the dependent variable and those considered scientifically relevant in other investigations [1,2,3,4,5,6,7,8,9,10,11,12,14,20]. In order to fit the multivariable model, the importance of each variable was verified. This included the examination of the Wald statistic for each variable and a comparison of each estimated coefficient with the coefficient from the univariate model containing only that variable. Variables that did not contribute to the model based on these criteria were eliminated and a new model was fitted. The new model was compared to the previous model using the Likelihood Ratio test. Furthermore, estimated coefficients for the remaining variables were compared to those from the full model. This process of deleting, refitting, and verifying continued until all the important variables are included in the model. The “enter modeling” method of STATA 14.0 was used for variable selection. Once the final model was obtained, the collinearity between variables was assessed by the variance inflation factor, and the interactions in the model analyzed.

The odds ratio (OR) and 95% confidence intervals (CI) were used to measure association. STATA software (StataCorp LP, College Station, TX, USA) was used for matching and analysis, with the statistical significance set to two-tailed, *p* < 0.05.

### 2.5. Ethical Aspects

For this investigation, we downloaded from Spanish Ministry of Health webpage the SNHIS2017 files [30]. Anyone can freely download this database, which, in order to guarantee confidentiality, is fully anonymized. According to Spanish legislation, the approval of an ethics committee was waived.

## 3. Results

**Figure 1:**
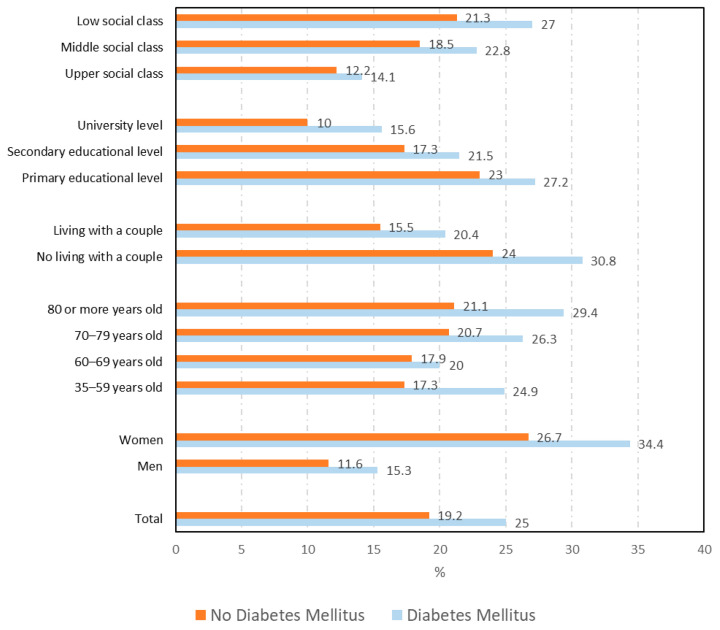
Prevalence of mental disorders according to sociodemographic variables among diabetes mellitus cases and controls.

**Figure 2:**
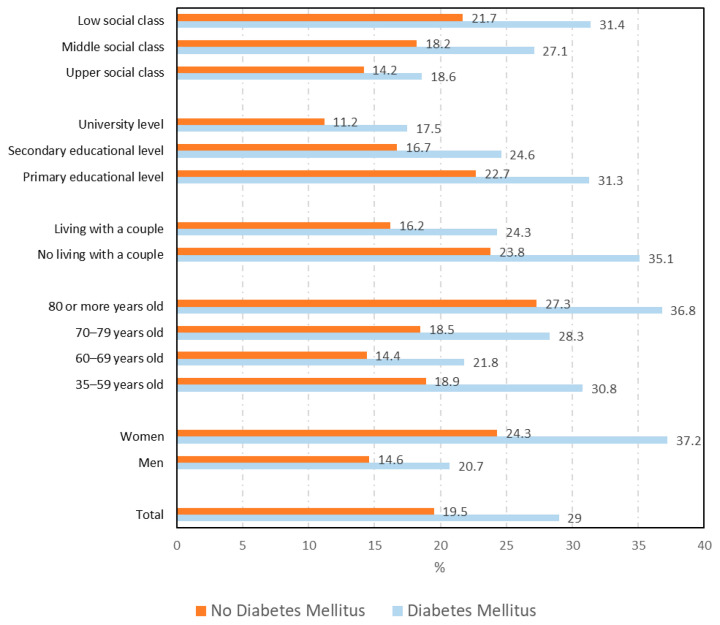
Prevalence of psychological distress according to sociodemographic variables among diabetes mellitus cases and controls.

**Figure 3:**
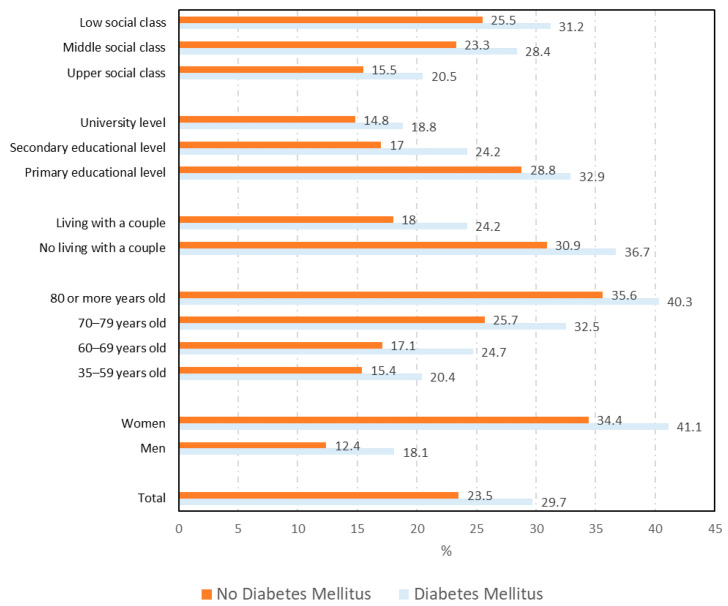
Prevalence of consumption of psychiatric medications according to sociodemographic variables among diabetes mellitus cases and controls.

The total number of DM cases that could be matched with a sex–age–province of residence control was 2116. As can be seen in Figure 1, Figure 2 and Figure 3, the overall prevalence of mental disorders (25.0% vs. 19.2%; *p* < 0.001), psychological distress (29% vs. 19.5%; *p* < 0.001), and consumption of psychiatric medications (29.7% vs. 23.5%; *p* < 0.001) among DM cases was higher than among matched non-DM controls.

The prevalence of the mental health variables according to sociodemographic variables among cases and controls are shown in Table S5 and Figure 1 for mental disorders, Figure 2 for psychological distress, and Figure 3 for the consumption of psychiatric medications.

As can be seen for the three variables analyzed, the prevalence among DM cases was significantly higher in all categories of most sociodemographic variables than among non-DM controls.

According to sex, the prevalence of mental disorders (34.4% vs. 26.7%; *p* < 0.001), psychological distress (37.2% vs. 24.3%; *p* < 0.001), and consumption of psychiatric medications (41.1% vs. 34.4%; *p* = 0.001) were higher among women with DM than among men with this condition. Also remarkable was that the highest prevalence was found among those DM cases with lower educational and social class levels for the three mental health variables. The prevalence of mental disorders according to health-related variables can be seen in Table 1. DM cases reported a higher prevalence of mental disorders than non-DM controls for all the categories of the variables shown in Table 1, with an exception made for the variable self-rated health and those who had a hospital admission in the last year, visited a psychologist in the last year, had a history of stroke, had an accident with permanent injuries, and/or were obese. According to the chronic conditions analyzed, the highest prevalence of mental disorders was found among DM cases who reported concomitant respiratory disease (40.9%), stroke (38.7%), or malignant tumors (37.2%).

Table S6 shows the results of the multivariable logistic regression to identify those variables independently associated with mental disorders among DM patients. Variables positively associated with reporting mental disorders included female sex (OR 1.66; 95% CI 1.18–2.33), worse self-rated health, a visit to a psychologist in the last year (OR 10.55; 95% CI 5.00–22.26), respiratory diseases, chronic pain, psychological distress (OR 3.17; 95% CI 2.32–4.35) and the consumption of psychiatric medications (OR 9.31; 95% CI 6.7–12.77). However, those in the oldest age group (80 years or over) (vs. 35–59 years, OR 0.56; 95% CI 0.32–0.98) and those who make more use healthcare resources such as a visit to the psychologist in the last year (OR 0.55; 95% CI 0.36–0.83) had a lower probability of mental disorders.

The prevalence of psychological distress among DM cases and controls according to health-related variables can be seen in Table 2. As reported for mental disorders, DM cases show higher prevalence than non-DM controls for most of the categories of the variables shown in Table 2. Non-significant differences were found for those who visited a psychologist in the last year and those who reported suffering from respiratory diseases or having had an accident that led to permanent injuries. The highest prevalence of psychological distress was found among DM cases with concomitant stroke (48.9%), malignant tumors (44.3%), or respiratory disease (39.6%).

The variables independently associated with reporting psychological distress among DM cases after multivariable analysis are shown in Table S7.

Women had a higher risk of psychological distress than men (OR 1.35; 95% CI 1.04–1.76). Older age groups (60–69 years, 70–79 years, and ≥80 years) had significantly lower probability than the youngest (age 35–59 years). 

Sociodemographic variables that increased the risk of psychological distress among DM cases included not living with a partner (OR 1.38; 95% CI 1.07–1.78). Cases with fair, bad, or very bad self-rated health were classified as experiencing psychological distress twice as often than those with a Very good/good self-rated health (OR 2.05; 95% CI 1.48–2.82).

The use of healthcare services in the previous year, such as emergency services or psychologist visits, were associated with presenting more psychological distress among DM cases.

Of the chronic conditions studied, a history of stroke was positively associated with psychological distress (OR 2.12; 95% CI 1.35–3.33). Regarding lifestyle variables, physical inactivity predicted less psychological distress (OR 0.56; 95% CI 0.43–0.72).

As expected, there were significant associations between psychological distress and mental disorders (OR 3.11; 95% CI 2.28–4.25) and consumption of psychiatric medications (OR 1.37; 95% CI 1.01–1.85).

The prevalence of self-reported consumption of psychiatric medications according to study variables is shown in Table 3. The prevalence was higher among DM cases than DM controls for most categories of the health variables analyzed. The highest prevalence among DM subjects was for those who visited a psychologist in the last year (81.8%), history of stroke (47.2%), and respiratory disease (42.9%).

The results of the multivariable analysis to identify which variables are independently associated with self-reported consumption of psychiatric medications among DM cases are shown in Table S8. 

Being a woman (OR 1.58; 95% CI 1.15–2.16) and older age are variables associated with higher consumption of psychiatric medications. Compared with the youngest age group, those aged 80 or over had a 2.18-fold higher probability of consuming these medications, and the risk increased linearly with age. Furthermore, worse self-rated health was positively associated with higher consumption of psychiatric medications (OR 1.95; 95% CI 1.36–2.79).

Regarding health-related variables, we observed that the use of health services in the previous year (emergency services or visit to psychologist) are risk factors for consumption of these drugs. Also, the presence of arthrosis, stroke, and/or chronic pain was significantly associated with the use of these medications.

Finally, there was a positive association between psychological distress (OR 1.34; 95% CI 0.99–1.83) and mental disorders (OR 9.26; 95% CI 6.76–12.67) with psychiatric medication utilization among DM cases.

## 4. Discussion

### 4.1. Main Findings

This study showed that people with DM have a higher prevalence of clinically diagnosed mental disorders, psychological distress, and consumption of psychiatric medications than subjects with the same sex, age, and province of residence without DM. Also, among those with DM, being a woman, worse self-rated health, and a visit to a psychologist were significantly correlated to the three mental health variables studied. 

We agree with previous investigations that have reported a higher prevalence of mental disorders and psychological distress in people with DM [1,5,6,9,16,20]. The results of the National Epidemiologic Survey on Alcohol and Related Conditions III, conducted in the USA, found mood disorders in 15.0% of DM subjects versus 13.3% among those without DM; for major depressive disorder, the equivalent prevalence was 11.5% versus 10.3% [16]. 

Prevalence estimates were higher in our study than those obtained in previous research using the SNHIS 2006, with values of 18.6% and 26% for mental disorders and psychological distress, respectively [20]. Observed increases in the prevalence of mental disorders and psychological distress among those with DM might be explained by the higher number of comorbid conditions in the DM population in 2017 compared with 2006 [20]; this has also been reported by other authors [31].

We found a greater difference in the prevalence for psychological distress (9.5%, from 29% to 19.5%) than for diagnosed mental disorders (6.8%, from 25.0% vs. 19.2%; *p* < 0.001) between subjects with and without DM. 

Self-reported use of physician-prescribed medications was significantly higher among subjects with DM. Keating et al. concluded that the higher prevalence of psychotropic drug prescription in patients with any type of DM may be due to the fact that an underlying psychological condition is expected to be present, such as a major depressive disorder [14]. 

### 4.2. Factors Associated with Mental Disorders, Psychological Distress (GHQ-12 ≥ 3), and Consumption of Psychiatric Medications

As found in most studies, women with DM had worse mental health, as measured with the three variables analyzed, than men with DM [2,3,20,32].

The association of bad self-rated health with mental disorders among DM patients has been reported before [5,20,32]. Experiencing mental disorders or psychological distress predicts poorer self-rated health and lower quality of life in persons with DM [5,20,32]. 

The greater use of medical and psychological services found in our investigation is in accordance with previous reports [6,14,20,33]. The effect of age on the prevalence of our dependent variables must be interpreted with caution. The mental health variables analyzed are related and therefore it is not correct to discuss them separately without considering their effect on each other. As expected and confirmed by the results in Tables S6 and S7, reporting mental disorders or psychological distress increases the probability of consumption of psychiatric medications. Previous works have described that, when an older person expresses feeling of sadness, agitation, or sleep problems, it is more likely that doctors will prescribe them psychiatric medications than those in younger age groups [34,35]. As a consequence of this higher prescription rate and the beneficial effect of these medications, the prevalence of older adults reporting mental disorders and psychological distress would decrease. Maust et al. analyzed data from elderly U.S. adults and demonstrated that the use of psychotropic drugs increased in primary care from 2003 to 2012. This suggests that patients in distress are seeking treatment and, given the growing public acceptance of psychotropic use, are increasingly willing to consider psychotropic medication. It may also indicate that, in general, physician prescriptions may be in response to mild or subsyndromal symptoms, with these patients obtaining more benefit from treatment and engagement with their providers [34]. A review of factors resulting in variations in prescribing rates showed that increased age was reported to lead to higher rates of antidepressant prescriptions [35].

The relationship between age and risk for mental disorders, in particular depression, in people with DM remains complicated and needs further exploration. Thus, some studies report lower rates of mental disorder in older individuals, while others report older age as a risk factor for higher prevalence [10,20,36,37,38].

The higher prevalence of mental disorders and psychological distress among younger age groups may be because dealing with an unexpected chronic condition such as DM requires an adaptation period and, during this period, it has been found that those diagnosed at younger ages cope less effectively with this new situation than older adults [39].

As previously reported, mental disorders, psychological distress, and the consumption of psychoactive drugs were associated with the presence of several comorbid chronic diseases in our study [17,40,41,42]. Fisher et al [17] concluded that, in people with DM, having more concomitant chronic comorbidities was significantly associated with a higher risk of suffering DM distress, anxiety, and depressive symptoms. Associations between DM or post-stroke depression and post-stroke dementia are well documented. Recently, Ouk et al. concluded that premorbid depression and DM are risk factors for developing dementia in the years after a stroke [41]. 

In terms of lifestyle, physical inactivity increased the probability of reporting psychological distress. The beneficial effect of physical exercise on mental health among DM patients has been reported before [20].

The relevance of identifying DM patients with mental disorders and psychological distress has been well established by previous works [4,5,6,7,8,9,10,11,12,13,14,15,20,43,44]. From a clinical point of view, it has been found that psychological distress in individuals with type 1 or type 2 diabetes, even at a level of severity below the threshold for a psychiatric diagnosis of anxiety or depression, is associated with poor glycemic control, poor adherence to treatment, poor control of cardiovascular risk factors (obesity, hypertension, dyslipidemia), higher rates of micro- and macro diabetes complications, and a decreased quality of life [4,5,6,7,8,9,10,11,12,13,14,15,20,43].

When the mental health comorbidities of DM are not diagnosed and treated, the financial cost to society and healthcare systems is substantial, as are the health consequences for patients [15]. Depression and DM result in unemployment and work disability as well as increased healthcare use and expenditure [15].

Besides the lower quality of life, DM and mental disorders specifically lead to increased social isolation [44,45]. Social isolation is increasingly appreciated as a risk factor for morbidity and mortality [46].

The American Diabetes Association and the International Diabetes Federation recommend that physicians “routinely screen for psychosocial problems such as depression and diabetes-related distress, anxiety, eating disorders, and cognitive impairment” [47,48]. These guidelines suggest that screening should be performed at the initial visit, at periodic intervals, and when there is a change in disease, treatment, or life circumstances [47,48]. This is extremely important as DM distress is only diagnosed and treated in about one-third of DM patients with these coexisting condition [15]. In our opinion, and in agreement with the international guidelines, all parts of the healthcare system (primary care, specialists, and hospital personal) should help to screen for mental disorders or DM distress [47,48]. However, in Spain primary care is the first point of call in the healthcare system regarding diabetes care, and thus the most important level for health prevention and promotion activities [16]. Therefore, intensive health services surveillance of these patients is necessary as multidisciplinary care has been proven to significantly improve emotional health and glucose control, reduce cardiovascular risk factors, prevent comorbidities and complications, and reduce medical care expenditure [15,33,43,44].

We think that our results may be useful for designing interventions to increase social interactions and reinforce the backing for social policies and local initiatives that attempt to enhance social support among middle-aged to older adults with and without DM with mental disorders and/or psychological distress. 

Finally, our findings can be translated into daily clinical practice: the presence of secondary complications and especially the persistence of poor glycemic control should alert doctors to the possibility of mental disorders, especially among women and the vulnerable elderly. In addition, enhanced efforts toward good glycemic control may contribute to improvements in mood and perceptions of well-being [49]. 

### 4.3. Strengths and Limitations 

The strengths of our research are the use of a population-based sample and a matched case–control design. The three variables used to assess mental health in our investigation are not independent as each component relates to the others. However, each provides additional information that complements the others and allows us to provide a wider vision of the effect of DM on mental health and establish comparisons with populations without DM or reporting other chronic conditions. Therefore, in our opinion, specific reasons that make it useful to evaluate these conditions separately include the following: (i) As mentioned before, almost 45% of participants with DM reported only one of these three conditions. (ii) The GHQ-12 is a multifactor screening tool, useful to detect mental disorders (depression/anxiety), social dysfunction, and loss of confidence [50]. People with DM experience distress as a consequence of their chronic condition and identifying them early is important to complement the information provided by other objective variables such as the diagnosis of depression and/or anxiety because it allows us to quantify a problem that may not have been detected by the healthcare system yet. Also, psychological distress has been proven to be useful as a measure of social support [50]. In fact, in our investigation, it was the only one of the three mental health variables analyzed that showed a significant association with not living with a partner. (iii) The analysis of psychiatric medication consumption provides additional information because the drugs analyzed include not only antidepressants and anxiolytic but also sleeping pills, providing information on sleeping disorders, which are not considered in the other two variables. This variable also provides information on the access to the healthcare system as we can use this variable to identify whether adults with mental disorders are being undertreated. iv) Several other investigations conducted in Spain have analyzed mental health using these three conditions separately, and we can therefore make direct comparisons [20,51,52,53].

However, our study has several limitations that are common to interview-based health surveys and have been previously reported [20,40,42,51,52,53]. First of all, not all the self-reported chronic conditions collected by the SNHIS have been validated against clinical records. However, in the case of DM a previous validation study reported a sensitivity for self-reported DM of over 70% and a specificity of over 95% [54]. Second, the SNHIS only collects information on the presence or absence of chronic conditions but not on the complications, duration, or severity of these conditions. Furthermore, data on the type of DM and the use of insulin or oral diabetic medications are not available. Third, as we use self-reported answers on clinical conditions that are not confirmed with medical records, the existence of a recall or social desirability bias cannot be ruled out. Fourth, the cross-sectional design makes it impossible to establish a causal relationship. Fifth, the SNHIS17 had a nonresponse rate of 27.8% [21], and this selection bias may affect our results. Sixth, in our investigation we analyzed the self-reported prevalence of depression and anxiety as a single condition, “mental disorders.” We did so because these two conditions appeared concomitantly with a very high frequency. For persons with DM, among those with self-reported anxiety (*n* = 269), as many as 195 (72.5%) also reported experiencing depression. The equivalent proportions for those without DM was 64.9%. With regard to depression, among persons with DM and depression (*n* = 402), 48.5% (*n* = 195) also reported anxiety. This proportion was 49.3% (147/298) in the controls without DM. Previous studies have reported that the overlap of depressive and anxiety disorders is quite common in DM patients [49]. This overlap makes the clinical and psychological management of these patients more difficult; due to the negative effects on health, the diseases should be diagnosed and treated simultaneously [49]. Seventh, we could not match 1.4% of subjects with DM included in the SNHIS17 and so they were excluded from the analysis. Given the very small proportion these subjects represent, in our opinion, the possible effect on our results is minimal.

## 5. Conclusions

The adults with DM included in our investigation had a significantly higher prevalence of mental disorders, psychological distress, and consumption of psychiatric medications than non-DM controls. It is necessary to implement screening strategies and psychological interventions to improve the mental health of DM patients in Spain, focusing especially on women and those aged 35 to 59 years. 

## Figures and Tables

**Table 1:** Prevalence of mental disorder according to health variables among subjects with DM and matched non-DM controls. (Spanish National Health Interview Survey, 2017.).

Health Variables	Categories	DM		Non-DM		
		*n*	%	*n*	%	*p*
Self-rated health	Very good/good	69	9.7	88	7.9	0.195
	Fair/poor/very poor	460	32.7	319	31.6	0.557
Emergency services in last year	Yes	266	32.8	154	25.9	0.005
	No	263	20.1	253	16.6	0.016
Hospital admission in last year	Yes	105	27.9	75	28.3	0.917
	No	424	24.3	332	17.9	<0.001
Visit to psychologist in last year	Yes	92	83.6	89	84.0	0.948
	No	437	21.8	317	15.8	<0.001
Hypertension	Yes	369	26.5	200	21.7	0.008
	No	160	22.1	207	17.3	0.010
Heart diseases	Yes	180	31.0	91	24.1	0.019
	No	349	22.7	316	18.2	0.001
Arthrosis	Yes	342	33.7	243	28.4	0.013
	No	187	17.0	164	13.0	0.007
Stroke	Yes	55	38.7	21	29.6	0.189
	No	474	24.0	386	18.9	<0.001
Respiratory disease	Yes	146	40.9	80	32.0	0.026
	No	383	21.7	327	17.5	0.001
Malignant tumor	Yes	71	37.2	49	27.2	0.041
	No	458	23.8	358	18.5	<0.001
Chronic pain	Yes	346	35.0	260	29.3	0.008
	No	183	16.2	147	11.9	0.003
Accident with permanent injuries	Yes	63	35.0	49	32.2	0.596
	No	466	24.0	358	18.2	<0.001
Obesity	Yes	171	25.9	89	23.0	0.299
	No	313	24.0	280	17.6	<0.001
Alcohol consumption in last 12 months	Yes	175	17.0	159	13.1	0.010
	No	354	32.5	248	27.4	0.013
Current smoking habit	Yes	82	25.5	64	18.8	0.039
	No	447	24.9	343	19.3	<0.001
Physical inactivity	Yes	325	31.5	220	25.8	0.007
	No	204	18.8	187	14.8	0.009

DM: Diabetes mellitus. *p*-value for comparison of prevalence between DM and matched non-DM subjects.

**Table 2:** Prevalence Psychological distress (GHQ-12 ≥ 3), according to health variables among subjects with DM and matched non-DM controls. (Spanish National Health Interview Survey, 2017.)

Health Variables	Categories	DM		Non-DM		
		*n*	%	*n*	%	*p*
Self-rated health	Very good/good	81	11.4	85	7.7	0.007
	Fair/poor/very poor	522	38.1	320	32.8	0.008
Emergency services in last year	Yes	322	40.9	176	30.7	<0.001
	No	281	21.8	229	15.2	<0.001
Hospital admission in last year	Yes	149	41.0	81	32.0	0.023
	No	454	26.5	324	17.7	<0.001
Visit to psychologist in last year	Yes	74	71.8	67	65.7	0.341
	No	529	26.8	338	17.1	<0.001
Hypertension	Yes	420	30.7	198	21.9	<0.001
	No	183	25.7	207	17.6	<0.001
Heart disease	Yes	217	38.3	94	25.7	<0.001
	No	386	25.5	311	18.1	<0.001
Arthrosis	Yes	378	38.3	222	26.4	<0.001
	No	225	20.6	183	14.8	<0.001
Stroke	Yes	68	48.9	20	32.3	0.032
	No	535	27.6	385	19.1	<0.001
Respiratory disease	Yes	137	39.6	83	34.6	0.218
	No	466	26.9	322	17.5	<0.001
Malignant tumors	Yes	82	44.3	51	29.3	0.003
	No	521	27.5	354	18.6	<0.001
Chronic pain	Yes	377	38.8	242	27.6	<0.001
	No	226	20.4	163	13.5	<0.001
Accident with permanent injuries	Yes	63	36.2	49	33.8	0.653
	No	540	28.3	356	18.4	<0.001
Obesity	Yes	209	32.2	85	22.2	<0.001
	No	343	26.6	291	18.6	0.001
Alcohol consumption in last 12 months	Yes	212	20.8	171	14.2	<0.001
	No	391	37.0	234	26.7	<0.001
Current smoking habit	Yes	95	29.6	61	18.0	<0.001
	No	508	28.9	344	19.8	<0.001
Physical inactivity	Yes	402	40.1	245	29.7	<0.001
	No	200	18.6	160	12.7	<0.001

DM: Diabetes mellitus. *p*-value for comparison of prevalence between DM and matched non-DM subjects.

**Table 3:** Prevalence of consumption of psychiatric medications according to health variables among subjects with DM and matched non-DM controls. (Spanish National Health Interview Survey, 2017.)

Health Variables	Categories	DM		Non-DM		
		*n*	%	*n*	%	*p*
Self-rated health	Very good/good	73	10.3	107	9.7	0.678
	Fair/poor/very poor	557	39.6	392	38.8	0.686
Emergency services in last year	Yes	334	41.2	201	33.8	0.005
	No	296	22.6	298	19.5	0.044
Hospital admission in last year	Yes	146	38.8	89	33.6	0.175
	No	484	27.8	410	22.1	<0.001
Visit to psychologist in last year	Yes	90	81.8	91	85.8	0.422
	No	540	26.9	408	20.3	<0.001
Hypertension	Yes	467	33.5	269	29.1	0.027
	No	163	22.5	230	19.2	0.087
Heart disease	Yes	236	40.6	133	35.2	0.091
	No	394	25.6	366	21.0	0.002
Arthrosis	Yes	431	42.4	318	37.1	0.018
	No	199	18.0	181	14.4	0.015
Stroke	Yes	67	47.2	32	45.1	0.771
	No	563	28.5	467	22.8	<0.001
Respiratory disease	Yes	153	42.9	88	35.2	0.058
	No	477	27.1	411	22.0	<0.001
Malignant tumors	Yes	80	41.9	57	31.5	0.038
	No	550	28.5	442	22.8	<0.001
Chronic pain	Yes	417	42.2	312	35.1	0.002
	No	67	47.2	32	45.1	0.771
Accident with permanent injuries	Yes	64	35.6	50	32.9	0.611
	No	566	29.2	449	22.8	<0.001
Obesity	Yes	206	31.1	99	25.6	0.057
	No	356	27.3	353	22.1	0.001
Alcohol consumption in last 12 months	Yes	216	21.0	195	16.1	0.003
	No	414	38.0	304	33.5	0.038
Current smoking habit	Yes	78	24.2	62	18.2	0.059
	No	552	30.7	437	24.6	<0.001
Physical inactivity	Yes	395	38.3	274	32.2	0.006
	No	235	21.7	225	17.8	0.017
Mental disorders	Yes	385	72.8	292	71.7	0.726
	No	244	15.4	206	12.0	0.006
Psychological distress (GHQ-12 ≥ 3)	Yes	324	53.7	209	51.6	0.507
	No	285	19.3	267	15.9	0.013

DM: Diabetes mellitus. *p*-value for comparison of prevalence between DM and matched non-DM subjects.

## Data Availability

This database can be downloaded freely and without cost from the website of the Ministry of Health, Social Services, and Equality (https://www.mscbs.gob.es/estadEstudios/estadisticas/encuestaNacional/encuesta2017.htm, accessed on 20 May 2021). All relevant data are reported within the paper.

## Supplementary Materials

The following are available online at https://www.mdpi.com/article/10.3390/ijerph18116088/s1, Table S1 [21,24]. Definition of variables used in our investigation according to the questions included in the Spanish National Health Interview Survey 2017. Table S2. Distribution according to the number of mental health variable among participants with diabetes mellitus and matched nondiabetic controls included in the Spanish National Health Interview Survey 2017. Table S3. Distribution according to the mental health variables analyzed among participants with diabetes mellitus included in the Spanish National Health Interview Survey 2017. Table S4. Distribution according to the mental health variables analyzed among participants with diabetes mellitus included in the Spanish National Health Interview Survey 2017. Table S5. Distribution of mental disorders, psychological distress (GHQ-12 ≥ 3), and consumption of psychiatric medications according to sociodemographic variables among subjects with DM and matched non-DM controls. Spanish National Health Interview Survey 2017. Table S6. Variables associated with the presence of mental disorders among persons experiencing DM. Spanish National Health Interview Survey 2017. Table S7. Variables associated with the presence of psychological distress (GHQ-12 ≥ 3) among persons experiencing DM. Spanish National Health Interview Survey 2017. Table S8. Variables associated with consumption of psychiatric medications among persons experiencing DM. Spanish National Health Interview Survey 2017.
