# Supplementary material for: Mental Health among Spanish Adults with Diabetes: Findings from a Population-Based Case–Controlled Study
Source: Int J Environ Res Public Health. 2021 Jun 4;18(11):6088. doi: 10.3390/ijerph18116088 (PMC8200218; doi:10.3390/ijerph18116088)
Supplement: Supplementary file 1 [file ijerph-18-06088-s001.zip › ijerph-1224637-supplementary.pdf]

Table S1. Definition of variables used in our investigation according to the questions included in the Spanish National Health Interview Survey 2017.

Questions	Description and Answer	Variables	Categories
12 items Global health questionnaire, (GHQ-12) [1,*]	The GHQ-12 consists of 12 items, each assessing the severity of a mental problem over the last few weeks using a 4-point Likert-type scale (from 0 to 3). Positive items were corrected from 0 (always) to 3 (never), and negative ones from 3 (always) to 0 (never). Scores on the GHQ-12 are obtained from a summation of responses to the 12 questions, with the first two response options scoring 0 and the last two scoring 1 (0–0–1–1).	Psychological distress GHQ-12 > 3	Yes: GHQ-12 > 3<br>No: GHQ-12 ≤3
- Next I am going to read you a list of types of medications, please tell me which one or more of them have you taken in the last 2 weeks?<br>- Was it prescribed by the doctor?	A list of 23 medications are read the person interviewed. For those medications with an affirmative answer in the first question, the second are completed consecutively for each specific medication.<br>1. Tranquilizers sedatives or "sleeping pills",<br>2. Antidepressants	Consumption of psychiatric medications	Yes: Those who answered "yes" to both question for options 1 and/or 2<br>No: Answered "no" to one or both questions for options 1 and 2
Which is your sex?	Men<br>Women	Sex	Men<br>Women
How old are you?	Age in years	Age groups	35–59 years<br>60–69 years<br>70–79 years<br>80 or more
Of the following, which better describes your current situation regarding who you live with?	1. Living with my husband/wife.<br>2. Living with my couple<br>3. Not living with a couple	Living with a couple	Yes. Options 1 and 2<br>No: Option 3
What level of education have you completed?	1. Does not know how to read or write<br>2. Incomplete primary education<br>3. Complete primary education (<br>4. First stage of Secondary Education, with or without a qualification<br>5. Elementary Spanish Upper Secondary Education])<br>6. Upper secondary education<br>7. Intermediate vocational training or equivalent<br>8. Advanced vocational training or equivalent<br>9. University studies or equivalent<br>10. Over university (master, PhD….)	Educational level	Primary Options 1 to 3<br>Secondary Options 4 to 8<br>University Options 9 and 10
The social class categories have been taken from the proposal made by the Spanish Society of Epidemiology's (SEE) Working Group on Determinants, in which social class is assigned according to occupation [2]	National Classification of Occupations 2011 (CNO2011), [2]	Social Class	Upper<br>Meddle<br>Lower

Questions	Description and Answer	Variables	Categories
In the past twelve month. How is your perception of your general health status?	1. Very good<br>2. Good,<br>3. Fair<br>4. Bad<br>5. Very bad.	Self-rated health	Very good/ good: Options 1 and 2<br>Fair/poor/very poor: Options 3 to5
In the last 12 months, have you had to use an emergency service for any problem or illness?	1. Yes<br>2. No	Emergency services in last year	1. Yes<br>2. No
In the last 12 months, have you had to be hospitalized for at least one night?	1. Yes<br>2. No	Hospital admission in last year	1. Yes<br>2. No
In the last 12 months, have you visited a psychologist?	1. Yes<br>2. No	Visit to psychologist in last year	1. Yes<br>2. No
1.Do you have or have you ever had any of the following diseases or heath conditions?<br>2. Have you suffered this disease/health condition in the past 12 months?<br>3. Was this disease/health condition been diagnosed by a doctor?	A card with a list of 32 conditions is shown to the person interviewed after the first question, and for those conditions reported by the participant, the second and third questions are completed consecutively for each specific condition.	• Chronic obstructive pulmonary disease<br>• Mental disorders (anxiety and/or depression)<br>• Hypertension<br>• Heart diseases (myocardial infarction, angina, coronary disease)<br>• Arthrosis,<br>• Stroke<br>• Diabetes mellitus<br>• Malignant tumours<br>• Chronic pain (neck pain and/or low back pain)<br>• Accident with permanent injuries	Yes: Answered affirmative to the three questions.<br>No: All the rest
1. Could you tell me how tall you are, approximately, without shoes?<br>2. Could you tell me your weight, approximately, without shoes and clothes?	Body mass index is calculated with the formulae:<br>Weight in kg/ (Height in meters)^2	Obesity	Yes: If Body mass index is 30 or over.<br>No: Yes: If Body mass index is under 30
During the past 12 months, how often have you had alcoholic beverages of any kind (i.e. beer, wine, spirits, distilled and mixed drinks, or other alcoholic beverages)	1. Daily or almost daily<br>2. 5–6 days per week<br>3. 3–4 days per week<br>4. 1–2 days per week<br>5. 2–3 days in a month<br>6. Once a month<br>7. Less than once a month<br>8. Not in the last 12 months, have I stopped drinking<br>9. Never or just a few sips to taste it throughout life	Alcohol consumption in last 12 months	Yes. Options 1 to 6<br>No: Option 7 to 9

Could you tell me if you smoke?	1. Yes, I smoke daily<br>2. Yes, I smoke, but not daily<br>3. I don't currently smoke but have smoked before<br>4. I neither smoke nor have I ever smoked regularly ..	Current smoking habit	Yes. Options 1 and 2<br>No: Option 3 and 4
Which of these possibilities best describes how often you do some physical activity in your free time?	1. I don't exercise. I occupy my free time almost completely sedentary.<br>2. I do some occasional physical or sports activity.<br>3. I do physical activity several times a month<br>4. I do sports or physical training several times a week	Physical inactivity	Yes. Option 1<br>No: Option 2 to 4

1. [1] Muñóz, P.E.; Vázquez, J.L.; Rodríguez Insausti, F.; Pastrana, E.; Varo, J. Adaptación española del General Health Questionnaire (G.H.Q.) de D. P. Goldberg (un método de identificación de casos psiquiátricos en la comunidad [Spanish adaptation of the General Health Questionnaire (G.H.Q.) of D. P. Goldberg (a method for identifying psychiatric cases in the community)]. *Arch. Neurobiol.* **1979**, *42*, 139–158. (Ref #24) [2] Instituto Nacional de Estadistica. Encuesta Nacional de Salud. 2017. Available online: https://www.ine.es/daco/daco42/clasificaciones/cno11_notas.pdfhttps://www.ine.es/en/metodologia/t15/t153041917_en.pdf (accessed on 12-February-2021). (Ref #21) *We grouped "fair" with "poor" and "very poor" instead of with "good" and "very good." because in our country "Fair", (in Spanish "regular"), has a negative connotation.

Table S2. Distribution according to the number of mental health variable among participant with diabetes mellitus and matched non-diabetes controls included in the Spanish National Health Interview Survey 2017.

Number of Mental Health Variable	Non-diabetes Controls			Diabetes Mellitus Cases		
	n	% Over Total	% *	*n*	% Over Total	% *
None	1352	63.8	NA	1133	53.5	NA
One	369	17.4	48.1	442	20.9	44.8
Two	252	11.9	32.9	312	14.7	31.6
Three	146	6.9	19.0	232	10.9	23.5

* Proportion over those with at least one mental condition. NA. Not applicable.

Table S3. Distribution according to the mental health variables analysed among participant with diabetes mellitus included in the Spanish National Health Interview Survey 2017.

Mental Health Variables	Mental Disorders, *n*(%)	Psychological Distress GHQ-12 > 3, *n*(%)	Consumption of Psychiatric Medications, *n*(%)	Total
Mental disorders, *n* (%)	NA	299 (56.5)	385 (72.5)	529
Psychological distress GHQ-12 > 3, *n* (%)	299 (56.5)	NA	324 (51.4)	603
Consumption of psychiatric medications. *n* (%)	385 (72.5)	324 (51.4)	NA	630
Total	529	603	630	NA

NA. Not applicable.

Table S4. Distribution according to the mental health variables analysed among participant with diabetes mellitus included in the Spanish National Health Interview Survey 2017.

Mental Health Variables	Mental Disorders, *n* (%)	Psychological Distress GHQ-12 > 3, *n* (%)	Consumption of Psychiatric Medications, *n* (%)	Total
Mental disorders, *n* (%)	NA	189 (46.2)	292 (71.2)	407
Psychological distress GHQ-12 > 3, *n* (%)	189 (46.2)	NA	209 (51.6)	405
Consumption of psychiatric medications. *n* (%)	292 (71.2)	209 (51.6)	NA	499
Total	407	405	499	NA

NA. Not applicable.

Table 5. Distribution of mental disorder, psychological distress (GHQ-12 ≥ 3) and consumption of psychiatric medications according to socio-demographic variables among subject with DM and matched non-DM controls. Spanish National Health Interview Survey 2017.

Socio-Demographic Variables		Mental disorders			Psychological distress GHQ-12 > 3			Consumption of psychiatric medications		
		DM	Non-DM	*p*	DM	Non-DM	*p*	DM	Non-DM	*p*
		n	*n*		*n*	*n*		*n*	*n*	
**Total**		529	407	<0.001	603	405	<0.001	630	499	<0.001
Sex	Men	160	121	0,01	213	150	<0.001	189	130	<0.001
	Women	369	286	<0.001	390	255	<0.001	441	369	0.001
Age groups	35–59 years	105	73	0.007	128	79	<0.001	86	65	0.059
	60–69 years	106	95	0.389	115	76	0.002	131	91	0.003
	70–79 years	172	135	0.016	183	120	<0.001	212	168	0.007
	80 or more	144	103	0.003	171	127	0.002	197	174	0.130
Living with a couple	No	288	220	0.001	318	213	<0.001	343	283	0.008
	Yes	241	187	0.002	285	192	<0.001	287	216	<0.001

Educational level	Primary	320	237	0.023	360	229	<0.001	387	298	0.042
	Secondary	143	123	0.044	163	118	<0.001	161	121	0.001
	University	24	29	0.092	27	32	0.078	29	43	0.282
Social class	Upper	29	41	0.513	38	47	0.178	42	52	0.140
	Middle	155	126	0.047	181	122	<0.001	193	159	0.031
	Low	314	219	0.002	258	219	<0.001	364	263	0.003

DM: Diabetes mellitus. *p* value for comparison of prevalence between DM and matched Non-DM subjects.

Table S6. Variables associated with the presence of mental disorders among persons experiencing DM. Spanish National Health Interview Survey 2017.

Health Variables	Categories	Odds Ratio	95% Confidence Interval
Sex	Men	1	
	Women	1.66	1.18–2.33
Age groups	35–59 years	1	
	60–69 years	0.73	0.45–1.18
	70–79 years	0.81	0.50–1.31
	80 or more	0.56	0.32–0.98
Self-rated health	Very good/ good	1	
	Fair/poor/very poor	1.61	1.09–2.38
Hospital admission in last year	No	1	
	Yes	0.55	0.36–0.83
Visit to psychologist in last year	No	1	
	Yes	10.55	5.00–22.26
Respiratory diseases	No	1	
	Yes	2.19	1.54–3.13
Chronic pain	No	1	
	Yes	1.42	1.02–1.96
Psychological distress (GHQ-12 ≥ 3)	No	1	
	Yes	3.17	2.32–4.35
Consumption of psychiatric medications	No	1	
	Yes	9.31	6.7–12.77

DM: Diabetes mellitus. *p* value for comparison of prevalence between DM and matched Non-DM subjects.

Table S7. Variables associated with the presence of Psychological distress (GHQ-12 ≥ 3), among persons experiencing DM. Spanish National Health Interview Survey 2017.

Health Variables	Categories	Odds Ratio	95% Confidence Interval
Sex	Men	1	
	Women	1.35	1.04–1.76
Age groups	35–59 years	1	
	60–69 years	0.49	0.33–0.72
	70–79 years	0.56	0.37–0.83
	80 or more	0.60	0.38–0.96
Living with a couple	Yes	1	
	No	1.38	1.07–1.78
Self-rated health	Very good/ good	1	
	Fair/poor/very poor	2.05	1.48–2.82
Emergency services in last year	No	1	
	Yes	1.38	1.06–1.80
Visit to psychologist in last year	No	1	
	Yes	2.07	1.17–3.64
Stroke	No	1	
	Yes	2.12	1.35–3.33

Physical inactivity	No	1	
	Yes	0.56	0.43–0.72
Consumption of psychiatric medications	No	1	
	Yes	1.37	1.01–1.85
Mental disorders	No	1	
	Yes	3.11	2.28–4.25

DM: Diabetes mellitus. *p* value for comparison of prevalence between DM and matched Non-DM subjects.

Table S8. Variables associated with consumption of psychiatric medications among persons experiencing DM. Spanish National Health Interview Survey 2017.

Health Variables	Categories	Odds Ratio	95% Confidence Interval
Sex	Men	1	
	Women	1.58	1.15–2.16
Age groups	35–59 years	1	
	60–69 years	1.61	1.01–2.56
	70–79 years	2.00	1.23–3.23
	80 or more	2.68	1.56–4.59
Self-rated health	Very good/ good	1	
	Fair/poor/very poor	1.95	1.36–2.79
Emergency services in last year	No	1	
	Yes	1.44	1.07–1.94
Visit to psychologist in last year	No	1	
	Yes	4.12	2.06–8.24
Arthrosis	No	1	
	Yes	1.49	1.08–2.04
Stroke	No	1	
	Yes	2.11	1.30–3.43
Chronic pain	No	1	
	Yes	1.66	1.23–2.23
Psychological distress (GHQ-12 ≥ 3)	No	1	
	Yes	1.34	0.99–1.83
Mental disorders	No	1	
	Yes	9.26	6.76–12.67

DM: Diabetes mellitus. *p* value for comparison of prevalence between DM and matched Non-DM subjects.
